# Revisiting olfactory receptors as putative drivers of cancer

**DOI:** 10.12688/wellcomeopenres.10646.1

**Published:** 2017-02-10

**Authors:** Marco Ranzani, Vivek Iyer, Ximena Ibarra-Soria, Martin Del Castillo Velasco-Herrera, Mathew Garnett, Darren Logan, David J. Adams

**Affiliations:** 1 Experimental Cancer Genetics, The Wellcome Trust Sanger Institute, Hinxton, UK; 2 Cancer Genome Project, The Wellcome Trust Sanger Institute, Hinxton, UK; 3 Genetics of Behaviour, The Wellcome Trust Sanger Institute, Hinxton, UK

**Keywords:** Olfactory receptor, cancer, survival signalling, proliferation, gene expression, melanoma, OR2C3

## Abstract

**BACKGROUND:** Olfactory receptors (ORs) recognize odorant molecules and activate a signal transduction pathway that ultimately leads to the perception of smell. This process also modulates the apoptotic cycle of olfactory sensory neurons in an olfactory receptor-specific manner. Recent reports indicate that some olfactory receptors are expressed in tissues other than the olfactory epithelium suggesting that they may have pleiotropic roles.

**METHODS:** We investigated the expression of 301 olfactory receptor genes in a comprehensive panel of 968 cancer cell lines.

**RESULTS:** Forty-nine per cent of cell lines show expression of at least one olfactory receptor gene. Some receptors display a broad pattern of expression across tumour types, while others were expressed in cell lines from a particular tissue. Additionally, most of the cancer cell lines expressing olfactory receptors express the effectors necessary for OR-mediated signal transduction. Remarkably, among cancer cell lines, *OR2C3* is exclusively expressed in melanoma lines. We also confirmed the expression of *OR2C3* in human melanomas, but not in normal melanocytes.

**CONCLUSIONS:** The pattern of *OR2C3* expression is suggestive of a functional role in the development and/or progression of melanoma. Some olfactory receptors may contribute to tumorigenesis.

## Abbreviations

OR = olfactory receptor; OSN = Olfactory sensory neuron; RSEM = RNA-Seq by Expectation-Maximization values; FPKM = fragment per kilobase per million mapped reads; RPKM = Reads Per Kilobase of transcript per Million mapped reads.

## Introduction

The olfactory receptors (ORs) represent the largest multi-gene family in the human genome and account for approximately 400 functional genes and 4–500 pseudogenes ^1, 2^. ORs encode G-protein-coupled receptor proteins that bind volatile molecules via their extracellular domain. Upon binding, they activate a signal transduction pathway that results in the generation of an electrical signal in olfactory sensory neurons (OSNs) which, after integration in the brain, results in the perception of smell ^1, 3^. This process involves the activation of a heterotrimeric G protein by the OR, that in turn activates adenylate cyclase resulting in the production of cyclic AMP (cAMP). cAMP acts as a second messenger that opens cyclic nucleotide gated channels which allow the depolarization of the membrane and the generation of an action potential in the activated OSN ^4^. In parallel, ligand binding also activates pathways involved in survival and proliferation ^5^, such as the MAPK, Rho and AKT signalling cascades ^6, 7^. Additionally, some ORs share high levels of identity with chemokine receptors and as such play a role in migration; indeed ORs mediate the projection of the OSN axons towards odorant-specific glomerular structures in the olfactory bulb ^8^.

ORs are frequently neglected in cancer genomic and transcriptomic studies because it is assumed that their specialised role in the olfactory epithelium makes them unlikely to contribute to cancer development. Nonetheless, their ability to sense small organic molecules, combined with the transduction of a survival signal and/or a migratory stimulus could render them functional in cancer cells ^9– 11^. In OSNs the ORs bind their ligand(s) in solution within the liquid layer of mucus ^12^, consequently they may be functionally triggered in other body fluids. Moreover, it has been shown that some ORs are expressed in tissues other than OSNs ^13, 14^ and a functional role for ORs has been demonstrated in sperm ^15^, muscle ^16^, kidney ^17^, carotid body ^18^ and lung ^19^. Recent studies have also shown that *OR51E2* (also known as *PSGR*) is involved in the regulation of prostate cancer cell migration and proliferation, and is a potential marker of patient outcome ^20– 22^. Therefore, we sought to evaluate the profile of OR expression in cancer cell lines and cancers.

## Methods

### Datasets and analysis

We investigated the expression of olfactory receptors in a broad panel of 968 cancer cell lines, using the RMA-normalised ^23^ microarray expression data available for each of 17,419 loci (annotation BrainArray v.10 ^24^). Data were available for each cell line in the deeply characterized COSMIC cell line collection (Iorio *et al.* ^25^). Information on these cancer cell lines is available from the COSMIC website: http://cancer.sanger.ac.uk/cell_lines. Raw expression data is publicly available via ArrayExpress (accession number: E-MTAB-3610; https://www.ebi.ac.uk/arrayexpress/experiments/E-MTAB-3610/).

**Figure 1.**
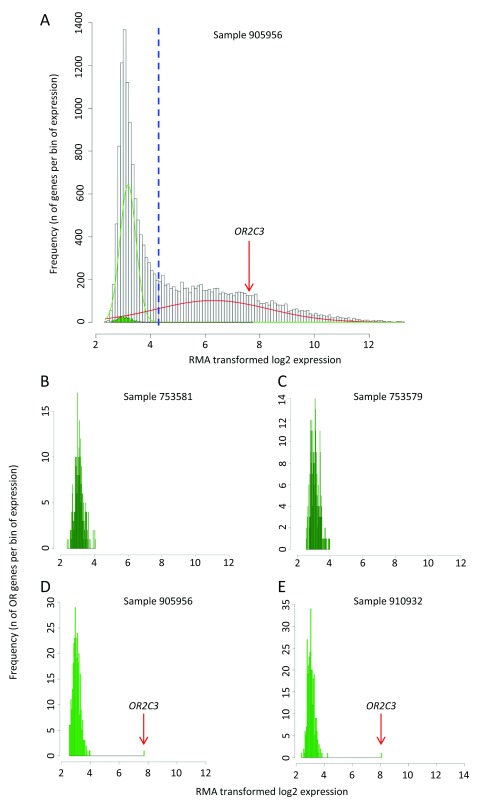
Olfactory receptor (OR) genes are expressed in cancer cell lines. **A**) Histogram representing the frequency (Y axis) of genes expressed per bin of gene expression (X axis: RMA normalised log2-transformed microarray expression data) for a representative melanoma cell line (SK-MEL-5 cell line, COSMIC ID 905956). The green bars represent the OR genes, the white bars all the other genes. The green and the red lines represent the modelling of the 2 peaks of the bimodal distribution. The dashed blue line shows the threshold of expression above which genes have the probability >95% to belong to the distribution of the expressed genes (right distribution in red). **B**– **E** Histograms represent the expression of OR gene in 4 representative melanoma cell lines (LB373-MEL-D, LB2518-MEL, SK-MEL-5, GAK with COSMIC IDs 753581, 753579, 905956, 910932, respectively), values displayed as in **A**. **B**– **C** do not show relevant expression of any OR gene, **D**– **E** display significant expression of *OR2C3* that is an outlier over the other ORs. The red arrow highlights *OR2C3* expression level.

**Figure 2.**
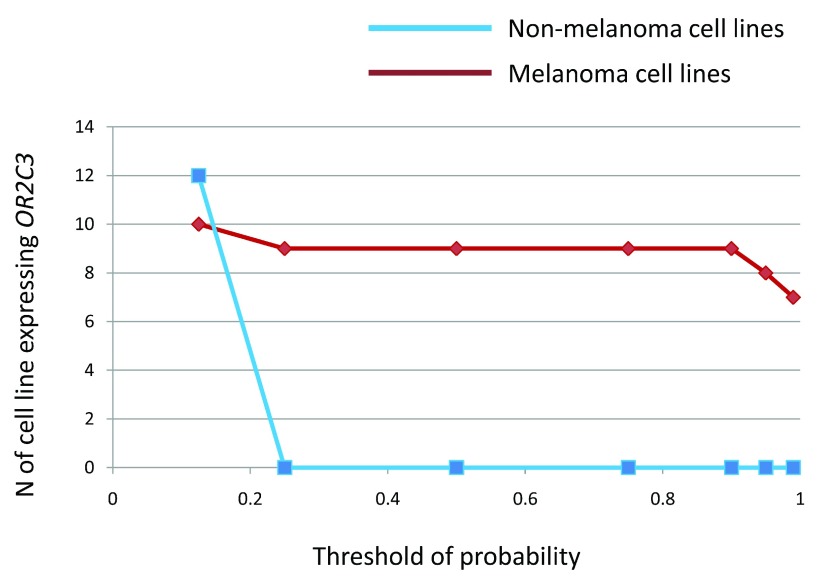
Frequency of *OR2C3* expression in cell lines with different thresholds of stringency. The Y axis shows the number of cell lines that express *OR2C3* at the threshold defined by X axis. On the X axis we showed the value of probability of belonging to the distribution of highly expressed genes (see Figure 1 and Methods) that we used as lower boundary threshold to define if *OR2C3* is expressed in each cell line. The blue line indicates non melanoma cell lines, the red line indicates melanoma cell lines. *OR2C3* is expressed only in melanoma cell lines till the threshold is dropped to 0.125. Number of expressing cell lines was calculated with thresholds >0.125, >0.25, >0.5, >0.75, >0.90, >0.95, >0.99.

To determine the level of expression of OR genes compared to the whole transcriptome, we applied mixture-distribution approaches described previously ^26, 27^. Briefly, we imported the cancer COSMIC cell line microarray data into R 3.2. We then binned the expression level of all the detected genes. By evaluating the frequency of genes included in each bin for an arbitrary cell line, we confirmed that the distribution of expression levels across the transcriptome was bimodal. Then for each cell line, we used the mixtools package (v1.0.4, available from CRAN) described in detail in 27 to perform an E-M (expectation-maximisation) algorithm decomposition of the observed bimodal distribution into two normal distributions. This approximated the classification of all genes into highly expressed and poorly/lowly expressed genes: note that the algorithm iteratively estimates *both* the mean and variance of each component distribution *and* the probability of any one observation of gene expression belonging to the “high” or “low” distributions. As an exemplar in Figure 1, we displayed the distribution of all gene-expression for cell line 905956, and plotted the algorithm-derived probabilities of any one gene belonging to low/high distributions using the mixtools “plot.EM” method. To create Supplementary Table 2, we noted the reported probabilities of any OR (and their effectors) belonging to the high or low distribution across all samples, where a gene was “highly expressed” if it had a 95% probability of belonging to the highly expressed geneset/distribution. For the *OR2C3* gene we performed a complementary analysis, where we varied the threshold probability needed to belong to the “highly expressed” geneset from 12.5% to 95%, and found the number of melanoma/non-melanoma cell lines with *OR2C3* as “highly expressed” ( Figure 2).

**Figure 3.**
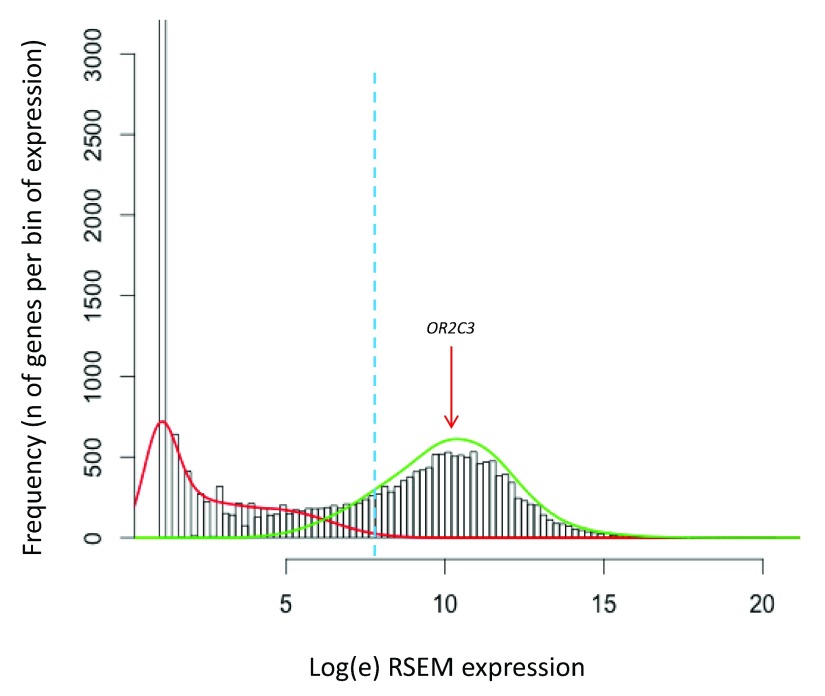
Distribution of gene expression in a representative melanoma sample. Histogram representing the frequency (Y axis) of genes expressed per bin of gene expression (X axis: expression levels are shown as [log _e_(RSEM+1)+1], see Methods, RSEM values from TCGA repository) for a representative melanoma tumour (TCGA.D9.A4Z6.06A.12R.A266.07). The green and the red lines represent the modelling of the 2 peaks of the bimodal distribution. The dashed blue line shows the threshold of expression above which genes have the probability >95% of belonging to the distribution of the expressed genes (right distribution in green).

The log-transformed RSEM (RNA-Seq by Expectation-Maximization)-normalised Illumina transcriptome sequencing data for human melanoma tumours ^28^, available via the TCGA website, were analysed for bimodality in the same way ( Figure 3). The results shown here are in whole or part based upon data generated by the TCGA Research Network: http://cancergenome.nih.gov/.

**Figure 4.**
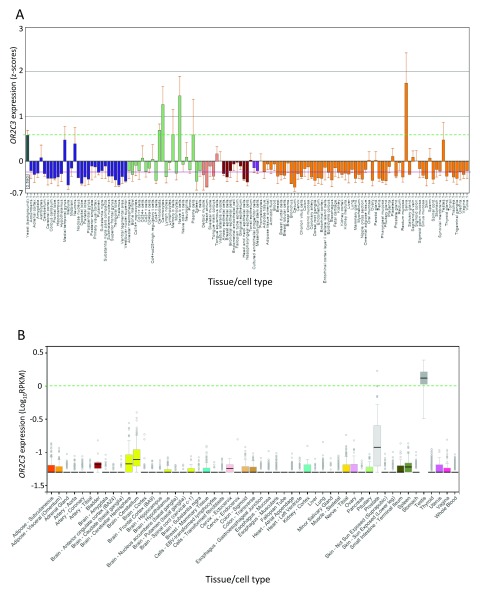
Expression of *OR2C3* in normal tissues. Output of the query for *OR2C3* in **A**) BioGPS and **B**) GTEx. The figure represents a screenshot of the output obtained interrogating the two databases for *OR2C3* expression. In **A**) BioGPS represents expression level from microarray as z-scores. A value >5 suggests that the gene is expressed in that tissue. As reference for background signal, the first column represents yeast RNA hybridization (value outlined with the green dashed line). None of the analysed tissues display expression of *OR2C3* (z-scores<5). In **B**) GTEx displays on the Y axis expression values in Log _10_ (RPKM) (Reads Per Kilobase of transcript per Million mapped reads). Box plots are shown as median and 25th and 75th percentiles; points are displayed as outliers if they are above or below 1.5 times the interquartile range. A very low expression level close to RPKM =1 (Log _10_ RPKM=0 in the figure, dashed green line) is observed only in testes.

We interrogated the expression of *OR2C3* in normal tissues from 2 repositories: BioGPS and GTEx. BioGPS contains expression levels from U133plus2 Affymetrix microarrays for 130 tissue types; the values shown on the Y axis are z-scores produced by the barcode function of the R package "frma" v1.0.0 ( http://www.bioconductor.org/packages/2.6/bioc/html/frma.html) ( Figure 4A). A z-score >5 suggests that the gene is expressed. As reference for the background signal, the first column represents yeast RNA hybridization (value outlined with the green dashed line). These data are available at: http://ds.biogps.org/?dataset=BDS_00001&gene=81472. GTEx Analysis Release v6 (dbGaP accession number: phs000424.v6.p1) shows expression values as log(RPKM) (Reads Per Kilobase of transcript per Million mapped reads) from 53 tissue types, calculated from a gene model with isoforms collapsed to a single gene ( Figure 4B). Box plots are shown as median and 25th and 75th percentiles; points are displayed as outliers if they are above or below 1.5 times the interquartile range.

We used data from the ENCODE database ( www.encodeproject.org; accession number: SRP039354) for the samples NHEM.f_M2_Rep1, NHEM.f_M2_Rep2, NHEM_M2_Rep1, NHEM_M2_Rep2. For the interrogation of *OR2C3* expression we used the expression values provided as fragment per kilobase per million mapped reads (FPKM).

We interrogated The Human Protein Atlas database ^29^ ( http://www.proteinatlas.org/) to investigate the expression of OR2C3 protein in melanoma tumor samples. By gene symbol we interrogated the protein expression of OR2C3 in different types of cancer.

## Results and discussion

Since the OR family represents around 3% of human genes and encodes proteins with the ability to transduce survival signals, we hypothesized that aberrant expression of ORs in cancer cells may contribute to cancer cell survival, proliferation and migration. To support the validity of this hypothesis, we investigated the expression of OR genes in a comprehensive panel of 968 cell lines from COSMIC ^30^. Overall we could interrogate the expression of 301 OR genes in this microarray gene expression dataset ( Supplementary Table 1). The distribution of expression levels across the transcriptome was found to be bimodal; when decomposed into two normal-like distributions, OR genes could be separated into those that were lowly expressed, and others that were highly expressed ^26, 27^ ( Figure 1A, green and red lines, respectively). OR genes were generally found to be expressed at low levels in cancer cell lines when compared to the distribution of expression levels of the whole transcriptome (see green bars in Figure 1A), with most of the cell lines displaying very low levels of expression of most OR genes (examples in Figure 1B–C). However, several cell lines displayed much higher expression of one or a few receptor genes that differentiate them from the other ORs, ranking these ORs amongst the set of genes defined as highly expressed when compared to the whole transcriptome (for instance *OR2C3* in Figure 1A, 1D–E). In order to define OR genes expressed at high levels in each cell line, we identified those ORs having a 95% or greater probability of belonging to the distribution of highly-expressed genes ( Supplementary Table 2 and Supplementary Table 3, see details of the approach in **Methods**). Overall, with this criterion, 56 (18.6%) OR genes are expressed in at least one cancer cell line, and 476 (49.2%) cell lines express at least one OR gene. Interestingly, 332 (69.7% of those expressing at least one OR) cell lines express only one specific receptor; this pattern of a single highly expressed OR was previously shown to be indicative of the functional receptor in an OSN ^31^. Some OR genes displayed high expression in several cell lines from different tumour types, such as *OR4A47*, *OR1D5*, *OR4C46* and *OR6B2*. Others ORs were primarily expressed in a single tumour type, such as *OR13A1* and *OR2C3* in B cell malignancies and melanoma, respectively ( Supplementary Table 2 and Supplementary Table 3). Additionally, we found that 85.95% of cell lines expressing an OR also express the functional effectors necessary for signal transduction (i.e. one of the alpha subunits of a G protein: *GNA15*, *GNA13*, *GNAO1*, *GNAI1*, *GNAL* and adenylate cyclase III *ADCY3*, see Supplementary Table 4). Therefore, most of the cell lines expressing an OR have the molecular machinery required to transduce signals from the OR to downstream pathways.

At the 95% threshold defined above, *OR2C3* is expressed in 8 out of 52 melanoma cell lines (15.4%, Supplementary Table 2), but not in any other line ( *P*<0.0001 by two-tailed Fisher’s exact test). These 8 melanoma cell lines also express OR effectors ( Supplementary Table 4). Remarkably, the specificity of *OR2C3* expression in melanoma cell lines is highly consistent since decreasing the probability threshold of OR expression still showed melanoma specific expression of *OR2C3* ( Figure 2), further supporting the validity of our analysis. *OR2C3* maps within an OR cluster located towards the telomeric end of chromosome 1 (1q44), and is not in the immediate vicinity of any established melanoma oncogenes whose deregulation/amplification could interfere with its expression.

To further explore the expression of *OR2C3* we interrogated a collection of human melanomas whose expression profile has been defined by RNA sequencing (TCGA ^28^, N=474 melanomas). We applied the same approach described above and found that 14 (3%) melanomas expressed *OR2C3* ( Supplementary Table 5 and Supplementary Table 6, and Figure 3). *OR2C3* expression in these melanomas is >130 in units of RNA-Seq by Expectation-Maximization values (RSEM). The difference in the frequency of *OR2C3* expression between cell lines and melanomas could be reflective of the different gene expression platforms used (microarrays vs Illumina transcriptome sequencing), or the analysis of melanoma sub-types that are not represented in the melanoma cell line collection. Additionally, we interrogated two gene expression repositories for normal tissue types (BioGPS ^32, 33^ and GTEx ^34^) and found that *OR2C3* is not expressed in any normal tissues (N=130 tissue types by BioGPS, N=53 tissue types by GTEx, Figure 4). Further, we found that *OR2C3* is not expressed in epidermal melanocyte samples from ENCODE (n=4, Supplementary Table 7), thus suggesting cancer-specific expression. We next interrogated The Human Protein Atlas database ^29^ for OR2C3 expression and found that 1 out of 11 melanoma samples express medium levels of OR2C3 ** protein. Together these results indicate the *OR2C3* is specifically expressed in a fraction of melanomas and supports a possible role for this gene in melanoma development.

## Conclusion

We conclude that, far from being just highly specialized components of the olfactory sub-genome, olfactory receptors should be considered as candidate cancer genes and warrant detailed functional investigation. In particular, we provided different lines of evidence that support a potential functional role for *OR2C3* in melanoma.

## Data availability

COSMIC cell line data: Raw expression data is publicly available via ArrayExpress (accession number: E-MTAB-3610; https://www.ebi.ac.uk/arrayexpress/experiments/E-MTAB-3610/.

The RSEM (RNA-Seq by Expectation-Maximization)-normalised Illumina transcriptome sequencing data for human melanoma tumours ^28^ is available via the TCGA website (project TCGA-SKCM; https://tcga-data.nci.nih.gov/docs/publications/tcga/, now hosted at https://gdc.cancer.gov/). The data are also available from: ftp://ftp.sanger.ac.uk/pub/users/vvi/tcga_rnaseq_v2_level3_skcm/tcga_sckm_rnaseqv2.tsv.gz.

BioGPS expression data (GEO accession number: GSE1133) is available at http://ds.biogps.org/?dataset=BDS_00001&gene=81472.

GTeX expression data for OR2C3 is available here: http://www.gtexportal.org/home/gene/OR2C3. The data were obtained from the GTEx Portal on 07/01/16.

Encode data is available here: www.encodeproject.org; accession number: SRP039354. 

Human Protein Atlas (version 16) data for all antibody HPA053920 melanoma samples is available here: http://www.proteinatlas.org/ENSG00000196242-OR2C3/cancer/tissue/melanoma

## Supplementary material

**Supplementary Table 1. List of COSMIC cell lines.** **A**) Complete list of COSMIC cell lines whose gene expression was interrogated. **B**) List of ORs that are detected by the microarray platform used to analyse gene expression for the COSMIC cell lines.

**Supplementary Table 2. Pattern of expression of olfactory receptor (OR) genes in cancer cell lines.** OR genes are displayed in columns, COSMIC cell lines are in rows with tissue and tumour type identifier. Values are the probability (from 0 to 1) of the OR gene to belong to the distribution of highly expressed genes (the right distribution represented in red line in Figure 1A). Only values above 0.95 (defined here as highly expressed) are shown and highlighted in red. Only cell lines with at least one OR above the threshold are shown, only ORs expressed above threshold in at least one cell line are shown. Full list of interrogated COSMIC cell lines and OR is presented in Supplementary Table 1A–B.

**Supplementary Table 3. Expression of ORs in cell lines summarized per tumour type.** The same data presented in Supplementary Table 2, here summarized per tumour type. The numbers represent cell lines expressing that specific receptor above the statistical threshold described in the text (>95%). Total number of cell lines per tumour type is also indicated.

**Supplementary Table 4. Expression of the OR effectors in cancer cell lines.** Genes are displayed in columns, COSMIC cell lines are in rows with tissue and tumour type identifiers shown. Values are the probability (from 0 to 1) of the OR effector genes belonging to the distribution of highly expressed genes. We show the expression of the key adenylate cyclase and the G alpha subunits associated with OR transduction. Expression of one G alpha subunit could be sufficient to transduce the signal. Only values above 0.95 are shown. Only cell lines that express adenylate cyclase 3 and one of the G alpha subunits are shown. The 8 melanoma cell lines expressing *OR2C3* are highlighted in green background.

**Supplementary Table 5. Pattern of expression of OR genes in human melanoma.** ORs are displayed in columns, melanoma samples with TCGA ID are displayed in rows. Values are the probability of the OR gene to belong to the distribution of expressed gene (the right distribution represented in red line in Supplementary Figure 2). Only values above 0.95 are shown and highlighted in red. Only melanomas with at least one OR above the threshold are shown, only ORs expressed above threshold in a melanoma are shown. Full list of interrogated melanomas and OR is shown in Supplementary Table 6. *OR2C3* is highlighted in yellow background. ORs detected in melanomas, but not measured in cell lines for the absence of probes in the microarray, are highlighted with a grey background. Data from TCGA.

**Supplementary Table 6. List of TCGA samples and OR genes interrogated.** The table includes the full list of melanoma samples analysed to generate Supplementary Table 5, and the full list of OR genes that were detected in the analysis of TCGA gene expression data.

**Supplementary Table 7. Expression level of *OR2C3* in primary melanocytes**. Table with the fragment per kilobase per million mapped reads (FPKM) values obtained for *OR2C3* in the ENCODE melanocyte samples NHEM.f_M2_Rep1, NHEM.f_M2_Rep2, NHEM_M2_Rep1, NHEM_M2_Rep2 (4 samples total).
