# Supplementary material for: Revisiting olfactory receptors as putative drivers of cancer
Source: Wellcome Open Res. 2017 Feb 10;2:9. [Version 1] doi: 10.12688/wellcomeopenres.10646.1 (PMC5421569; doi:10.12688/wellcomeopenres.10646.1)
Supplement: Peer review history

# Peer review: Referee response for version 1

**DOI:** 10.21956/wellcomeopenres.11472.r21999

**Published:** 2017-05-02

**Authors:** Daniela Biziato

**Affiliations:** 1 Cell and Gene Therapy Catapult, London, UK

In this work, Ranzani and colleagues report an extensive analysis of the expression of olfactory receptors (ORs) and elements of their downstream signaling cascades in cancer cell lines. Interestingly, they have found that about half of cancer cell lines express at least one OR gene and most of them express only one specific receptor. Some OR genes are highly expressed across different tumor types whilst others are highly expressed in a single specific tumor type. OR2C3 in particular, which was not previously related to cancer, was found highly expressed in several melanoma cell lines but not in other cancer cell lines or in normal melanocytes.

 The data in the manuscript are convincing, substantiating and expanding the role of ORs as potential target or biomarkers in cancer.

 However several ORs, such as OR51E1, OR51E2 and OR2W3, which have been previously described to be upregulated in cancer tissues (1-5) do not emerge as highly expressed ORs in the analysis reported this manuscript. A discussion on different methodologies used in different studies and the utility of cell lines as tumor models would make the article scientifically sound.

 As stated in the paper, only 1 out of 11 melanoma samples reported in the Human Protein Atlas database express OR2C3 protein. An independent analysis of OR2C3 protein expression in a higher number of samples would provide further robustness to the data reported.

I have read this submission. I believe that I have an appropriate level of expertise to confirm that it is of an acceptable scientific standard.

## References

[1] CuiTTsolakisAVLiSCCunninghamJLLindTÖbergKGiandomenicoV: Olfactory receptor 51E1 protein as a potential novel tissue biomarker for small intestine neuroendocrine carcinomas. *Eur J Endocrinol*.2013;168(2) : 10.1530/EJE-12-0814 253-61 10.1530/EJE-12-0814 23184910

[2] LejaJEssaghirAEssandMWesterKObergKTöttermanTHLloydRVasmatzisGDemoulinJBGiandomenicoV: Novel markers for enterochromaffin cells and gastrointestinal neuroendocrine carcinomas. *Mod Pathol*.2009;22(2) : 10.1038/modpathol.2008.174 261-72 10.1038/modpathol.2008.174 18953328

[3] GiandomenicoVCuiTGrimeliusLÖbergKPelosiGTsolakisAV: Olfactory receptor 51E1 as a novel target for diagnosis in somatostatin receptor-negative lung carcinoids. *J Mol Endocrinol*.2013;51(3) : 10.1530/JME-13-0144 277-86 10.1530/JME-13-0144 23969981

[4] XuLLSunCPetrovicsGMakaremMFurusatoBZhangWSesterhennIAMcLeodDGSunLMoulJWSrivastavaS: Quantitative expression profile of PSGR in prostate cancer. *Prostate Cancer Prostatic Dis*.2006;9(1) : 10.1038/sj.pcan.4500836 56-61 10.1038/sj.pcan.4500836 16231015

[5] ZhouBIrwantoAGuoYMBeiJXWuQChenGZhangTPLeiJJFengQSChenLZLiuJZhaoYP: Exome sequencing and digital PCR analyses reveal novel mutated genes related to the metastasis of pancreatic ductal adenocarcinoma. *Cancer Biol Ther*.2012;13(10) : 10.4161/cbt.20839 871-9 10.4161/cbt.20839 22797009

# Peer review: Referee response for version 1

**DOI:** 10.21956/wellcomeopenres.11472.r20133

**Published:** 2017-02-27

**Authors:** David M. Lin

**Affiliations:** 1 Department of Biomedical Sciences, Cornell University, Ithaca, NY, USA

The authors have completed a scientifically sound analysis of olfactory receptor expression, with a suitably complete explanation of the study design, methods, and datasets. The authors are to be commended for the breadth of their analysis. Suggestions for improvement are as follows:

1. The most obvious concern is the lack of independent validation of their analysis (e.g. qRT-PCR, in situ hybridization, IHC, etc.). A possible alternative would be to ask whether their analysis detects olfactory receptors previously shown to be expressed in certain cancer types. These include 51E2 (cited by the authors as an impetus for these studies), 2AT4 (leukemia), 51E1 (lung, gastric, neuroendocrine), OR2W3 (pancreatic), and others. All of these receptors were detected by the authors, but their expression patterns (Supplemental Table 1) do not always match published expression patterns. A discussion describing the sensitivity and/or limitations of their analysis would be helpful.
2. The authors ignore the trace amino acid receptors (TAARs), which are also considered olfactory receptors. While the expression of family members outside of the olfactory epithelium is thought to be limited (with the exception of TAAR1), it would have been interesting to know if they detected any such expression in cancer cells. At a minimum, they should note why they excluded these receptors from their analysis. MS4As have also recently been implicated in odorant sensing, but given the recent nature of this discovery, it is not surprising that they were not included in this work.
3. The authors assert that olfactory receptor expression in cancer has essentially been neglected in cancer research. This statement is in part in the eye of the beholder, however, multiple papers have been published recently showing functional analyses of olfactory receptor signaling on cancer progression. The authors should consider expanding their citations. Some of these studies are listed below.
4. In the abstract, the authors state that "recent" reports indicate the presence of non-epithelial olfactory expression. But such expression is hardly recent, and has been known since the late 1990's, as I'm sure the authors are aware.  

I have read this submission. I believe that I have an appropriate level of expertise to confirm that it is of an acceptable scientific standard.

## References

[1] GelisLJovancevicNVeitingerSMandalBArndtHDNeuhausEMHattH: Functional Characterization of the Odorant Receptor 51E2 in Human Melanocytes. *J Biol Chem*.2016;291(34) : 10.1074/jbc.M116.734517 17772-86 10.1074/jbc.M116.734517 27226631PMC5016170

[2] SanzGLerayIGrébertDAntoineSAcquistapaceAMuscatABoukadiriAMirLM: Structurally related odorant ligands of the olfactory receptor OR51E2 differentially promote metastasis emergence and tumor growth. *Oncotarget*.2017;8(3) : 10.18632/oncotarget.13836 4330-4341 10.18632/oncotarget.13836 28032594PMC5354835

[3] ManteniotisSWojcikSBrauhoffPMöllmannMPetersenLGöthertJRSchmiegelWDührsenUGisselmannGHattH: Functional characterization of the ectopically expressed olfactory receptor 2AT4 in human myelogenous leukemia. *Cell Death Discov*.2016;2: 10.1038/cddiscovery.2015.70 15070 10.1038/cddiscovery.2015.70 27551494PMC4979481

[4] MoritaRHirohashiYTorigoeTIto-InodaSTakahashiAMariyaTAsanumaHTamuraYTsukaharaTKanasekiTKuboTKutomiGMizuguchiTTeruiTIshitaniKHashinoSKondoTMinagawaNTakahashiNTaketomiATodoSAsakaMSatoN: Olfactory Receptor Family 7 Subfamily C Member 1 Is a Novel Marker of Colon Cancer-Initiating Cells and Is a Potent Target of Immunotherapy. *Clin Cancer Res*.2016;22(13) : 10.1158/1078-0432.CCR-15-1709 3298-309 10.1158/1078-0432.CCR-15-1709 26861454
